# Within-Session Sequence of the Tennis Serve Training in Youth Elite Players

**DOI:** 10.3390/ijerph18010244

**Published:** 2020-12-31

**Authors:** Jaime Fernandez-Fernandez, Manuel Moya-Ramon, Francisco Javier Santos-Rosa, Petrus Gantois, Fábio Yuzo Nakamura, David Sanz-Rivas, Urs Granacher

**Affiliations:** 1 Department of Physical Activity and Sport Sciences, Universidad de León, 24071 León, Spain; jaime.fernandez@unileon.es; 2 AMRED, Human Movement and Sports Performance Analysis, Universidad de León, 24071 León, Spain; 3 Tennis Research Group, 28080 Madrid, Spain; dsanzrivas@gmail.com; 4 Department of Sports Sciences, Miguel Hernandez University, 03202 Elche, Spain; mmoya@umh.es; 5 Faculty of Sport, Pablo de Olavide University, 41013 Seville, Spain; fjsantos@upo.es; 6 Associate Graduate Program in Physical Education, Federal University of Paraiba, João Pessoa 58051-900, Brazil; pgm.gantois@gmail.com (P.G.); fabioy_nakamura@yahoo.com.br (F.Y.N.); 7 Research Center in Sports Sciences, Health Sciences and Human Development (CIDESD), University Institute of Maia (ISMAI), 4475-690 Maia, Portugal; 8 Division of Training and Movement Sciences, Research Focus Cognition Sciences, University of Potsdam, 14469 Potsdam, Germany

**Keywords:** athletes, athletic performance, fatigue, fitness, shoulder strength, range of motion

## Abstract

The influence of muscular fatigue on tennis serve performance within regular training sessions is unclear. Therefore, the aim of the present study was to examine the within-session sequence of the tennis serve in youth tennis. Twenty-five young male (14.9 ± 0.9 years) and female (14.5 ± 0.9 years) players participated in this within-subject crossover study, and they were randomly but sex-matched assigned to different training sequences (serve exercise before tennis training (BTS) or after tennis training (ATS)). Pre- and post-tests included serve velocity performance and accuracy, shoulder strength, and range-of-motion (ROM) performance (internal/external rotation). Results showed that after one week of serve training conducted following the ATS sequence, significant decreases were found in serve performance (e.g., speed and accuracy), with standardized differences ranging from *d* = 0.29 to 1.13, as well as the shoulder function (strength [*d* = 0.20 to 1.0] and ROM [*d* = 0.17 to 0.31]) in both female and male players, compared to the BTS sequence. Based on the present findings, it appears more effective to implement serve training before the regular tennis training in youth players. If applied after training, excessive levels of fatigue may cause shoulder imbalances that could be related to an increased injury risk.

## 1. Introduction

Tennis is an intermittent sport in which players entail a mixture of physical components, such as linear sprint and change-of-direction speed, agility, muscle power, and cardiovascular fitness in order to achieve high levels of performance [1]. Players are required to execute high amounts of strokes per training/match with powerful shots including serves and groundstrokes [2,3]. Particularly, the serve plays an important role in tennis match outcome, allowing the player to win the point directly through an ace or dominate the rally right from the start [4].

During the early stages of long-term athlete development (i.e., under 14 players (U14)), players spend a great amount of training time mastering their individual tennis-specific skills, with technical and tactical sessions often exceeding 15–20 h per week [5]. Additionally, daily training practices of young tennis players often involve undertaking inadequately scheduled training sessions conducted within close proximity. As a consequence, players have to conduct short and non-appropriate warm-up protocols [1]. These warm-up protocols normally involve 8–10 min of general exercises, followed by shoulder strengthening routines (i.e., using elastic bands), ground strokes, volleys, and low-intensity smashes. Thereafter, players perform ~40–50 min of tennis-specific drills (i.e., mixed open/closed technical/tactical drills). This is normally followed by serve training for another 10 min [6].

Although the demands in tennis are multifactorial, including physical, psychological, technical, and tactical attributes [7], coaches and players themselves often associate the final outcome of a match with a decline in hitting accuracy and/or performance/fitness due to fatigue. In this regard, fatigue can be considered the exercise-induced transient reduction in the force generating capacity of a muscle [8]. Although it is unclear to what extent players experience fatigue during regular training sessions, few studies have reported the negative effects of fatigue on stroke performance [7,9]. Of note, there is information available from previous studies that serve and groundstroke velocity declined with the progression of tennis matches or after completion of the matches [10,11,12].

As evidenced above, the serve represents a highly important stroke in tennis and fatigue may lead to a decline in stroke quality and efficiency. Accordingly, a reduced serve velocity could negatively affect tennis performance, especially in young tennis players. Moreover, as a result of the repetitive demands induced by continuous practice and play, tennis players are susceptible to a range of injuries including acute traumatic injuries and chronic overuse conditions [13]. In this regard, the tennis serve provokes physical stress to the shoulder joint [14], leading to a considerable prevalence of overuse injuries in elite junior tennis players [15,16]. These injuries are primarily caused by the conjunction of unilateral and repeated tennis strokes, and biomechanical, training and competition workloads, which lead to altered shoulder range of motion (ROM) and imbalanced muscle strength [17]. In fact, it can be hypothesized that fatigue may even increase the risk of sustaining injuries during the serve [18]. Based on these assumptions, the within-session sequence of serve training during tennis sessions is important from two perspectives. First, there is evidence that motor skill learning should be conducted in an unfatigued condition (i.e., at the beginning of the session) to achieve better outcomes [19,20]. Second, the risk of sustaining injuries may increase if serve training is conducted at the end of the session.

Thus, the aim of the present study was to investigate the within-session sequence of the tennis serve in youth tennis. We hypothesized that training the serve at the end of the regular tennis session would cause acute performance declines in the form of serve speed and a higher fatigued state of the shoulder function (e.g., strength and ROM) compared to training the serve at the beginning of the tennis session [1,21].

## 2. Materials and Methods

### 2.1. Study Design

**Figure 1:**
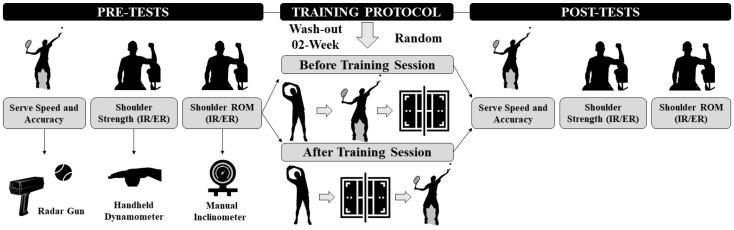
Experimental protocol.

The study was performed at the beginning of the summer competition season (April–May). A within-subject crossover study was designed in which participants were randomly assigned to both interventions (e.g., serve exercise before tennis training (BTS) or after tennis training (ATS)). Simple and sex-matched randomization was carried out using a website tool (www.randomizer.org). Prior to the start of the study, participants were familiarized with the study procedures and assessment routines. Tests were always completed on the same day for BTS and ATS. Four days before the first test, all players participated in a pilot session. Thereafter, four test sessions were scheduled (Figure 1). After the first test session (Pre-Test 1), participants performed a regular training week, including 4 days of tennis-specific training and 2 fitness sessions. During the tennis-specific sessions, participants completed the sessions with the following sequence: standardized warm-up, serve training, and tennis training (if players were assigned to the BTS condition). At the end of the week, participants were tested again (Post-Test 1). Two weeks later (wash-out period), participants completed a 3rd test session (Pre-Test 2) and conducted a similar training week, but the tennis-specific sessions were conducted as follows: standardized warm-up, tennis training, and serve training (ATS). A 4th test session (Post-Test 2) was conducted after this second training week. The group that started the first experimental training week with ATS finished the second experimental training week with BTS. The participants had a 3-day rest after Post-Test 1. Thereafter they resumed their regular training routine. In order to not repeat one of the training programs (BTS or ATS), the tennis serve was conducted in isolated sessions. In addition, training volume was significantly reduced with only 2 serve sessions during the washout period.

Pre- and post-BTS and ATS tests were conducted for the assessment of serve velocity (SV) performance and accuracy (ACC), shoulder strength, and range-of-motion (ROM) performance (i.e., internal/external rotation). All fitness tests were performed on an indoor synthetic court and a physiotherapy room at the same time of day. A 24-h recovery was granted between the last training session and the post-tests. To minimize possible biases effects from uncontrolled variables, all players were instructed to maintain their habitual lifestyle and normal dietary intake before and during the investigation. They were told to attend all experimental conditions in a well-rested state prior each test and to consume their last (caffeine-free) meal at least 24 h before the scheduled test time.

### 2.2. Participants

Twenty-five young male (*n* = 12; 14.9 ± 0.9 years; body mass 59.1 ± 6.6 kg, body height 172.5 ± 7.0 cm; maturity offset (MO) 0.8 ± 1.0) and female (*n* = 13; 14.5 ± 0.9 years; body mass 55.3 ± 7.0 kg, body height 163.5 ± 6.5 cm; MO 2.0 ± 0.8) tennis players participated in this study (Table 1). Twenty-one players were right-handed and four were left-handed. Participants had a mean training background of 7.5 ± 1.2 years and participated, on average, in 8–10 h of weekly tennis training, focused on the development of on-court technical/tactical tennis skills, plus two weekly days of ~1 h fitness training. Players were free from severe injuries, did not have surgeries, did not conduct any sport-related rehabilitation during the 6 months prior to the commencement of the study, and were ranked among the 200 best players in their respective national singles ranking category (U15–U16). Due to organizational limitations in the tennis clubs, individual counterbalance was not possible, although pre-tests were used to control the initial status of players. Written informed consent was obtained prior to the start of the study from all players and their parents/legal guardians. All participants were fully informed about the study procedures. The study was approved by the Institutional Ethics Review Committee (Spanish Tennis Federation; RFET_CE17.3) and conformed to the code of ethics of the World Medical Association (Declaration of Helsinki).

### 2.3. Testing Procedures

#### 2.3.1. Maturity Status

Body height was measured with a fixed stadiometer to the nearest 0.1 cm (Holtain Ltd., Crosswell, UK), sitting height using a purpose-built table (±0.1 cm; Holtain Ltd., Crosswell, UK), and body mass with a digital scale to the nearest 0.1 kg (ADE Electronic Column Scales, Hamburg, Germany) [22]. Pubertal timing was estimated according to the maturity offset method, as previously described [23]. The age of peak linear growth (age at peak-height velocity (PHV)—APHV) is an indicator of somatic maturity representing the time of maximal growth in stature during adolescence [24]. Maturity level (in years) was calculated by subtracting the chronological age at the time of assessment from the chronological peak velocity age. Thus, tennis players were classified as follow: “−1.0” (1 year before his PHV); “0” (at the time of his PHV); or “+1” (1 year after his PHV) [25].

#### 2.3.2. Serve Speed and Accuracy

Serve velocity was recorded using a radar gun (Stalker Professional Sports Radar, Richardson, TX, USA). This procedure followed previous recommendations [10,26]. Briefly, the radar gun was positioned to face the center of the baseline, 3 m behind the server, and aligned with the approximate height of ball contact (~2.2 m) and pointing down the center of the court. A standardized specific serve warm-up was carried out for each player. It consisted of 5 min of upper body mobility and two sets of first and second serves (8 repetitions each). Thereafter, players performed 3 sets of maximum speed serves. All serves were executed on the deuce side of the court. Players used their own racquets and a set of new balls which were provided (Babolat Gold, Lyon, France) for the test. To be able to record data, the serves had to land within 1 m of the center service line. The highest serve speed recorded was used for subsequent statistical analyses. The inter-day reliability for this test ranged from 0.90 to 0.98 (intraclass correlation coefficients (ICCs) values).

**Figure 2:**
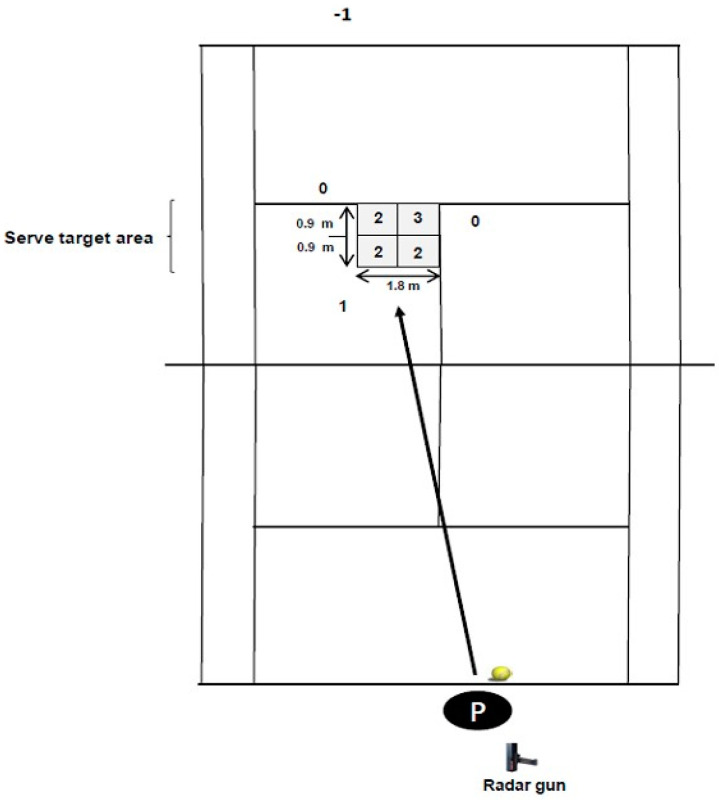
Representation of the serve performance test and target area dimensions. P: Player.

For serve accuracy, players performed another 5 maximum effort serves with approximate the same spin (flat or slice) to the “advantage” service box. The serve accuracy was determined by the number of times the balls landed within a selected target area according to a 3, 2, 1, scoring system as showed in Figure 2. Shots landing outside the target areas (i.e., errors) received a “0” score. The target area for the serve (1.8 m × 1.8 m) was inside the intersection of the service line and the center line. Participants served from the deuce court and were instructed to “serve first serves flat and down the T” (center line). These procedures are well-accepted as valid measure of tennis serve accuracy by players, coaches, and scientists [3], and were previously adopted in male junior tennis players [26].

#### 2.3.3. Shoulder Range-of-Motion (ROM)

Dominant and non-dominant passive glenohumeral rotation were assessed with a manual inclinometer (ISOMED inclinometer, Portland, Oregon) as previously described [27]. Briefly, each player laid down in supine position on a bench with the shoulder 90° abducted and the elbow flexed to 90° (forearm perpendicular to the bench). From this starting position, an examiner held the participant’s proximal shoulder region (i.e., clavicle and scapula) against the bench to stabilize the scapula by avoiding an overpressure. Another examiner rotated the humerus in the glenohumeral joint to produce maximum passive external (ER) and internal (IR) rotation [28]. Two trials of IR and ER-ROM on each shoulder were performed and the average performance (°) was used for statistical analysis. Moreover, the total range-of-motion (TROM; sum of IR and ER) was calculated. Inter-day reliability ranged between 0.94 to 0.99 (ICCs values).

#### 2.3.4. Shoulder Strength

Isometric shoulder IR and ER strength of the dominant and the non-dominant side were assessed with a portable handheld dynamometer (Nicholas Manual Muscle Tester, Lafayette Indiana Instruments, Lafayette, IN, USA) as previously described [29]. Players were in supine lying position on a plinth with the shoulder abducted at 90° and the elbow flexed at 90°. Two attempts were performed (5 s) for shoulder IR and ER strength on each side and the average values was recorded for analysis. Each trial was interspersed by a 30 s passive rest period. In addition, shoulder IR and ER strength performance were normalized to body mass and expressed as N/kg [30]. ICCs values ranged from 0.91 to 0.98.

#### 2.3.5. Tennis Training Sessions

The training programs were always conducted on outdoor tennis courts between 17:00 and 19:30 h at the same facility. During the two weeks of intervention, all players, males and females, exercised in 3–4 groups, with similar goals. In addition to the regular tennis training (4 times per week), all participants performed twice weekly neuromuscular training, as fitness sessions, for the duration of the study, following recently published recommendations [29,31]. These sessions were always conducted before the tennis-specific training sessions. If necessary, a 10–15 min rest time was allowed between training bouts, during which players were able to consume water and a 6% carbohydrate/electrolyte drink ad libitum. Regular tennis training lasted on average 65.2 ± 5.2 min and was characterized by a ~8-min specific warm-up (i.e., including ground strokes, volleys, and low-intensity smashes), ~10–15 min of serve training for the BTS condition, and ~45–50 min of specific drills (i.e., mixed open/closed technical/tactical drills). For the ATS condition the tennis-training sequence was standardized warm-up, specific drills and serve training. In most cases, the tennis training session of this study was designed by the tennis instructors with the goal of developing the specific needs of each player, including more technical/tactical drills (i.e., designed to focus on improvements to a specific quality in stroke technique or tactical approach) and/or sessions including a more physical approach (i.e., relatively high volumes of open and/or high-intensity drills) [32,33]. Regarding the serve training volume, a similar number of serves was conducted during BTS (i.e., 72.5 ± 17.1 balls) and ATS (72.5 ± 5.0 balls) sessions [4].

### 2.4. Statistical Analyses

Data were presented as means and standard deviations. Normal distribution and homogeneity of data were assessed and confirmed using the Shapiro–Wilk and the Levene test, respectively. A two-way analysis of variance (ANOVA) was computed with the factors time (pre- and post-training) and condition (before tennis and after tennis training) for repeated measures. If time-by-condition interactions reached the level of significance, Bonferroni corrected post hoc tests were computed to identify pairwise differences. The effect size of the ANOVA model (partial eta square—η^2^) was used to judge the practical significance. In addition, we calculated the relative changes of each outcome (Δ% = ([post−pre]/pre)*100) along with within-subjects’ effect size (ES). Threshold values for Cohen’s d ES statistics were 0.2 (small), 0.5 (moderate), and 0.8 (large) [34]. The intraclass correlation coefficients (ICCs) and their 95% confidence interval (based on the single measure, absolute-agreement, and 2-way mixed effects model), the standard error of measurement $(\mathrm{SEM}=\mathrm{SDpre}\;\times \;\sqrt{1-\mathrm{ICC}}$), and minimal detectable change $\left(\mathrm{MDC}=1.96\;\times \;\mathrm{SEM}\;\times \;\sqrt{2}\right)$ were calculated to assess the reliability of dependent variables measures [35]. The analyses were performed using the statistical package for the social sciences (SPSS, IBM^®^ corporation, Chicago, IL, USA), version 20.0, with a significance level of 5%.

## 3. Results

Table 1 shows participants’ characteristics according to sex. Results indicated differences between males and females for age at peak-height velocity (PHV) (t_(23)_ = −7.5; *p* < 0.001), MO (t_(23)_ = 3.3; *p* = 0.003), and body height (t_(23)_ = −3.2; *p* = 0.004).

Table 2 shows reliability data for all analyzed variables. Overall, high-degrees of reliability were found for each dependent variable (ICCs ranging from 0.957 to 0.991; excellent), with the exception of serve accuracy that showed a moderate reliability (ICC = 0.674). In addition, overall low values were found for the standard error of measurement (SEM), ranging from 1.61 to 6.75.

### 3.1. Serve Speed and Accuracy Performance

**Figure 3:**
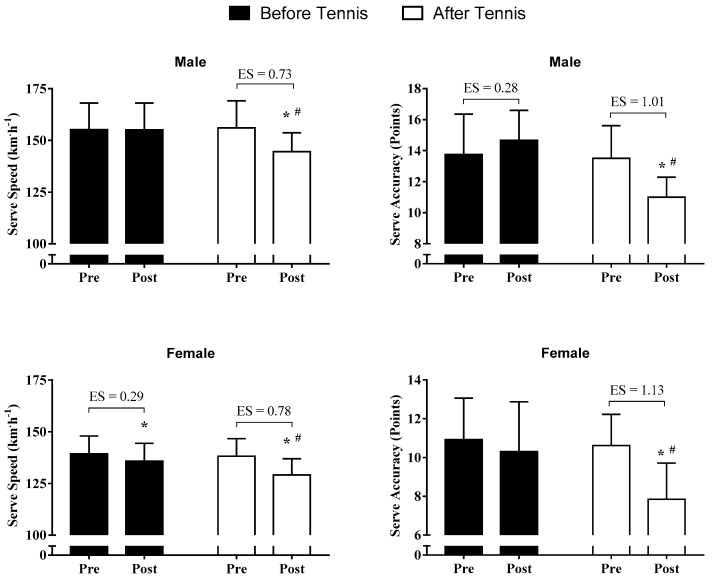
Serve speed and accuracy performance at baseline and after the intervention (before or after tennis training session) according to sex; * = main effect of time (*p* < 0.05); # = time-by-condition interaction effect (*p* < 0.05).

Results showed a significant main effect of time for serve speed performance in males (F_(1,11)_ = 46.2; *p* < 0.001; η^2^ = 0.81) and females (F(_1,12_) = 41.2; *p* < 0.01; η^2^ = 0.77). Moreover, a significant time-by-condition interaction was found for both males (F_(1,11)_ = 45.1; *p* < 0.001; η^2^ = 0.80) and females (F_(1,11)_ = 7.9; *p* = 0.16; η^2^ = 0.40). Specifically, post hoc analyses revealed that serve speed performance was compromised in ATS for males (*p* < 0.001; 95% IC = 7.66 to 13.34; Δ% = −7.7% and ES = 0.73 [moderate]) and females (*p* = 0.016; 95% IC = 3.26 to 10.09; Δ% = −6.5% and ES = 0.78 [moderate]) compared with BTS (males Δ% = 0% and females Δ% = −2.1% and ES = 0.29 [small]) (Figure 3).

Regarding serve accuracy, the analysis revealed a time-by-condition interaction for males (F_(1,11)_ = 35.9; *p* < 0.001; η^2^ = 0.76) and females (F_(1,12)_ = 10.7; *p* = 0.07; η^2^ = 0.47). Specifically, serve accuracy was compromised in the ATS condition for both males (BTS Δ% = 7.2% vs. ATS Δ% = −18.5% and ES = 1.01 [large]) and females, in contrast to BTS (BTS Δ% = −5.5% vs. ATS Δ% = −25.9% and ES = 1.13 [large]). Moreover, a significant main effect of time was found for females (F_(1,12)_ = 23.8; *p* < 0.01; η^2^ = 0.66), but not for males (F_(1,11)_ = 2.5; *p* = 0.143; η^2^ = 0.18).

### 3.2. Shoulder Strength

#### 3.2.1. Males

There was a significant main effect of time for all, absolute and normalized, shoulder strength measures (*p* < 0.05; η^2^ ranging from 0.420 to 0.830). Moreover, a significant time-by-condition interaction effect was observed for absolute (F_(1,11)_ = 34.2; *p* < 0.001; η^2^ = 0.76) and normalized (F_(1,11)_ = 34.7; *p* < 0.001; η^2^ = 0.76) shoulder IR strength of the dominant side, absolute (F_(1,11)_ = 10.8; *p* = 0.007; η^2^ = 0.50) and normalized (F_(1,11)_ = 11.8; *p* = 0.006; η^2^ = 0.52) IR strength of the non-dominant side, as well as absolute (F_(1,11)_ = 5.1; *p* = 0.046; η^2^ = 0.32) and normalized (F_(1,11)_ = 5.6; *p* = 0.038; η^2^ = 0.34) ER strength of the non-dominant side. Specifically, post hoc analyses revealed lower absolute and normalized shoulder strength in the ATS compared to BTS (BTS = Δ% ranging from 0% to −6.5% with an ES ranging from 0 to 0.20 [trivial to small]; ATS = Δ% ranging from −4.5% to −16.9% with an ES ranging from 0.15 to 1.00 [trivial to large]) (Table 3).

#### 3.2.2. Females

The statistical analyses revealed a significant main effect of time for absolute (F_(1,12)_ = 72.3; *p* < 0.001; η^2^ = 0.86) and normalized (F_(1,12)_ = 77.9; *p* < 0.001; η^2^ = 0.87) IR strength of the dominant side, absolute (F_(1,12)_ = 6.0; *p* = 0.030; η^2^ = 0.33) and normalized (F_(1,12)_ = 5.3; *p* = 0.040; η^2^ = 0.31) IR strength of the non-dominant side, and absolute (F_(1,12)_ = 10.4; *p* = 0.007; η^2^ = 0.46) and normalized (F_(1,12)_ = 10.5; *p* = 0.007; η^2^ = 0.47) ER strength of the dominant side. In addition, a significant time-by-condition interaction was found for the absolute (F_(1,12)_ = 28.4; *p* < 0.001; η^2^ = 0.70) and normalized (F_(1,12)_ = 29.5; *p* < 0.001; η^2^ = 0.71) IR strength of the dominant side, as well as for the normalized (F_(1,12)_ = 5.4; *p* = 0.038; η^2^ = 0.31) ER strength of the dominant side. Post hoc analyses revealed lower shoulder, absolute, and normalized, strength in ATS compared with BTS (BTS = Δ% ranging from 0% to −7.4% with an ES ranging from 0 to 0.35 [trivial to small]; ATS = Δ% ranging from −5.9% to −20% with an ES ranging from 0.35 to 1.00 [small to large]) (Table 4).

### 3.3. Shoulder Range of Motion (ROM)

#### 3.3.1. Males

A significant main effect of time was found for IR ROM of the dominant side (F_(1,11)_ = 236.9; *p* < 0.001; η^2^ = 0.96) and non-dominant side (F_(1,11)_ = 21.2; *p* = 0.001; η^2^ = 0.66), ER ROM of the dominant side (F_(1,11)_ = 111.5; *p* < 0.001; η^2^ = 0.91), and shoulder TROM of the non-dominant side (F_(1,11)_ = 5.1; *p* = 0.045; η^2^ = 0.32). Moreover, a significant time-by-condition interaction was observed for IR (F_(1,11)_ = 15.8; *p* = 0.002; η^2^ = 0.59) and ER ROM (F_(1,11)_ = 6.1; *p* = 0.032; η^2^ = 0.35) of the dominant side. Specifically, lower IR ROM values were found for the dominant side following ATS in contrast to BTS (BTS Δ% = −5.7% and ES = 0.17 [trivial] vs. ATS Δ% = −8.3% and ES = 0.26 [small]). On the other hand, higher ER ROM values were observed for the dominant side in ATS compared with BTS (BTS Δ% = 2.7% and ES = 0.25 [small] vs. ATS Δ% = 3.6% and ES = 0.31 [small]).

#### 3.3.2. Females

Results showed a main effect of time for IR (F_(1,12)_ = 56.9; *p* < 0.001; η^2^ = 0.83), ER ROM (F_(1,12)_ = 75.0; *p* < 0.001; η^2^ = 0.86), and TROM (F_(1,12)_ = 9.1; *p* = 0.011; η^2^ = 0.43) of the dominant side. In addition, a significant time-by-condition interaction was found for the IR (F_(1,12)_ = 49.5; *p* < 0.001; η^2^ = 0.80) and ER (F_(1,12)_ = 18.4; *p* = 0.001; η^2^ = 0.60) ROM of the dominant side, and IR ROM of the non-dominant side (F_(1,12)_ = 6.9; *p* = 0.022; η^2^ = 0.37). Post hoc analyses showed lower IR ROM on the dominant side for ATS compared with BTS (BTS Δ% = −2.0% and ES = 0.08 [trivial] vs. ATS Δ% = −6.0% and ES = 0.25 [small]), whereas a lower IR ROM was observed in the non-dominant side in BTS compared with ATS (BTS Δ% = −1.5% and ES = 0.05 [trivial] vs. ATS Δ% = −0.3% and ES = 0.01 [trivial]). In addition, higher shoulder ER ROM was found on the dominant side for ATS compared with BTS (BTS Δ% = 2.3% and ES = 0.18 [trivial] vs. ATS Δ% = 3.6% and ES = 0.30 [small]).

## 4. Discussion

To the best of our knowledge, this is the first study analyzing the within-session sequence of the tennis serve in youth tennis players. After one week of serve training conducted at the end of the regular tennis sessions (ATS), significant decreases were found in serve performance (e.g., speed) as well as the shoulder function (e.g., strength and ROM) in both female and male players, compared to the training protocol with serve training scheduled at the beginning of the tennis session (BTS).

Although there is a lack of specific research regarding the training loads associated with the serve in tennis, during a regular training session or match, players usually hit between 50 and 150 serves [9,36,37]. However, this number depends on the competitive level, age, and/or gender. In this regard, the serve has commonly been related to an increased risk of sustaining injuries, primarily due to the repetitive large ROM, multi-joint loading, and rotational speeds which are required during stroke performance [36,37]. The reported serve volumes in this study are in agreement with previous research analyzing the serve volume of junior tennis players [4,38,39], and cannot be considered to present an overload for healthy players.

Regarding the serve performance (e.g., speed and accuracy), results of this study showed that the ATS condition resulted in significant decreases in speed (~−8%) and accuracy (−18.5 to −26%) in both males and females. The lack of studies analyzing the within sequence effects of serve training prelude comparisons. We can speculate that the observed reductions could be related to neuromuscular, physiological, and/or biomechanical factors. For instance, exercise-induced muscle damage due to the high eccentric component during serve performance, and more specifically, during the deceleration of the arm, might compromise muscle function and thus the capacity to produce power [7]. Moreover, the performance of tennis sessions prior to the serve could impair the capacity to produce power due to fatigue. This could affect speed and accuracy during serve training when compared to an unfatigued state, as in the BTS condition. In this regard, the present data are in line with previous studies showing that acute muscle fatigue might be harmful to serve performance (i.e., speed (−4.5%) and accuracy (values ranging from −12% to 30%)) in well-trained tennis players [11,40,41]. Unfortunately, specific fatigue-related measures (i.e., post-creatine kinase concentrations, muscle soreness, or perceived exertion) were not reported and should be examined in future studies.

Interestingly, our data showed that in females, serve speed was reduced in BTS and ATS (Figure 2). This suggests the inclusion of serve-specific sessions during the training week and/or to combine serve training with different strength training sessions.

Serve speed, as previously mentioned, has been reported to be the most powerful, potentially dominant stroke in tennis [4], and it relies on several factors, including upper-body strength/power and shoulder ROM [3]. Our results showed that, although both conditions (BTS and ATS) resulted in decreased shoulder strength (Table 2 and Table 3), the ATS condition was accompanied by significantly higher decreases in the strength levels on the dominant side (i.e., IR: from −3.5% to −6.8%, ER: −2.4 to −6.5). Previous studies analyzing IR–ER maximal strength levels of the shoulder after prolonged matches [42], consecutive matches on the same day [31], or consecutive days of prolonged match play [43] reported similar acute reductions in levels of shoulder strength (−6–8%). These strength reductions lead to a less effective use of the stretch shortening cycle in the shoulder rotators during the cocking and acceleration phases [9], and consequently, to a decrease in serve performance, as reflected by a ~7% decrease in speed and a 17–29% decrease in accuracy following ATS. Thus, and with reference to widely reported definitions of fatigue in the literature [8], we postulate that upper-body strength was affected by fatigue, especially in the ATS condition.

Previous research suggested that a reduction in shoulder ROM increases the likelihood of shoulder injuries in racket sports [44], although results are conflicting. Thus, an excessive or limited shoulder ROM may contribute to shoulder pathologies such as instability and impingement [45]. The results of this study showed that, after a training week with the ATS condition compared with BTS, IR ROM significantly decreased on the dominant side in males (−8.3%) and females (−6%). Moreover, a significantly higher ER on the dominant side was also observed in ATS compared with BTS condition for males (+3.6%) and females (3.6%). Again, as there is no research analyzing the within sequence effects of serve training, we had to compare our data with studies analyzing the acute effects of several matches on the same day or prolonged consecutive matches on the shoulder ROM [31,32,42]. These studies reported larger changes in ROM values, with deficits ranging from 4 to 20%.

Although previous research showed that injured overhead athletes have impairments in shoulder ROM compared with uninjured athletes [43,45], the reported ROM alterations may represent a normal adaptation due to the greater hitting demands and are therefore, not associated with an increased risk of sustaining shoulder injuries. Moreover, it has recently been suggested that ROM screening may not be effective to identify overhead athletes (i.e., handball, volleyball, or tennis) who are at risk of sustaining shoulder and elbow injuries [45].

As in any other sport, tennis training is sometimes based on old beliefs and anecdotal evidence from coaches, lacking scientific support. In this regard, the tennis serve has been traditionally included at the end of the regular tennis sessions. However, based on our findings, it appears more effective to implement serve training before the regular tennis training in youth players. If applied after training, excessive levels of fatigue may cause shoulder imbalances that could be related to an increased injury risk. Moreover, from a training perspective, the application of the BTS within-session sequence could be beneficial to induce improvements in serve performance. Finally, alterations in the shoulder function suggest the importance of including a shoulder profile assessment in the physical testing of tennis players as well as to monitor shoulder function throughout the competitive season.

A number of study limitations are worth mentioning. Although chronological age of the players was similar, MO differed significantly. This is most likely due to the fact that males and females were examined at similar chronological age. Thus, more research is needed to analyze males and females of different maturity levels (i.e., pre-, around, and post-PHV). Due to the lack of information regarding the loads associated with serve training in both training and competition, we had to speculate as to the applied training volumes. More research is required to investigate how different players respond to habitual short and long-term training routines. We are confident that the present study shows high levels of ecological validity and may offer a starting point to suggest practical applications to strength and conditioning as well as to tennis training.

## 5. Conclusions

In conclusion, the present study showed that training the serve at the end of the regular tennis sessions (ATS) compared with a protocol conducted at the beginning of the session (BTS) resulted in significant decreases in serve performance (e.g., speed) together with impaired shoulder function (e.g., strength and ROM) in female and male players.

## Figures and Tables

**Table 1:** Participants’ characteristics according to sex (means and standard deviations).

Variables	Males (*n* = 12)	Females (*n* = 13)	*p*-Value
Chronological age (years)	14.9 ± 0.9	14.5 ± 0.9	0.335
APHV (years)	14.2 ± 0.5	12.6 ± 0.5	<0.001
MO (years)	0.8 ± 1.0	2.0 ± 0.8	0.003
Height (cm)	172.5 ± 7.0	163.8 ± 6.5	0.004
Body mass (kg)	59.1 ± 6.6	55.3 ± 7.0	0.179

APHV = age at peak-height velocity; MO = maturity offset.

**Table 2:** Test-retest reliability for all analyzed performance measures.

95% Confident Interval					
Variables	ICC	Lower Bound	Upper Bound	SEM	MDC
Serve Speed	0.957	0.905	0.981	2.72	7.53
Serve Accuracy	0.674	0.387	0.842	2.52	3.83
**Dominant Side**					
IR Strength	0.960	0.913	0.982	6.75	18.72
ER Strength	0.969	0.927	0.987	4.84	13.41
IR ROM	0.990	0.953	0,997	1.61	4.49
ER ROM	0.986	0.943	0.995	1.83	5.08
**Non-Dominant Side**					
IR Strength	0.979	0.953	0.991	4.60	12.74
ER Strength	0.979	0.954	0.991	3.15	8.74
IR ROM	0.991	0.951	0.997	1.92	5.33
ER ROM	0.986	0.925	0.995	1.74	4.81

ICC = intra-class correlation coefficient; SEM = standard error of measurement; MDC = minimal detectable change; IR = shoulder internal rotation; ER = shoulder external rotation; ROM = range of motion.

**Table 3:** Data of male players showing shoulder strength and range of motion (ROM) measures at baseline and before or after the tennis training session.

	Before Tennis				After Tennis				*p*-Value
Variables	Pre	Post	Δ%	Pre	Post	Δ%	Time Effects	Condition Effects	Interaction Effects
**Shoulder Strength**									
IR D Strength (N·m^−1^)	179.3 ± 35.3	173.1 ± 30.1	−3.5	179.7 ± 33.9	149.4 ± 24.6	−16.9	<0.001	0.003	<0.001
IR D Strength (N·m^−1^·kg^−1^)	3.0 ± 0.4	2.9 ± 0.3	−3.3	3.0 ± 0.4	2.5 ± 0.3	−16.7	<0.001	0.001	<0.001
ER D Strength (N·m^−1^)	121.2 ± 27.0	113.32 ± 23.5	−6.5	125.5 ± 24.4	113.0 ± 23.1	−10.0	<0.001	0.082	0.216
ER D Strength (N·m^−1^·kg^−1^)	2.0 ± 0.4	1.9 ± 0.4	−5.0	2.1 ± 0.3	1.9 ± 0.3	−9.5	<0.001	0.340	0.096
IR ND Strength (N·m^−1^)	154.0 ± 31.7	149.7 ± 31.4	−2.8	152.1 ± 33.0	141.5 ± 29.9	−7.0	<0.001	0.055	0.007
IR ND Strength (N·m^−1^·kg^−1^)	2.6 ± 0.5	2.5 ± 0.5	−3.8	2.6 ± 0.5	2.4 ± 0.5	−7.7	<0.001	0.063	0.006
ER ND Strength (N·m^−1^)	117.8 ± 23.3	116.6 ± 21.4	−1.0	114.5 ± 23.5	109.4 ± 24.1	−4.5	0.017	0.002	0.046
ER ND Strength (N·m^−1^·kg^−1^)	2.0 ± 0.3	2.0 ± 0.3	0	1.9 ± 0.3	1.8 ± 0.3	−5.3	0.017	0.001	0.038
**Shoulder ROM**									
IR D (°)	56.0 ± 12.9	52.8 ± 13.2	−5.7	56.7 ± 13.0	52.0 ± 13.0	−8.3	<0.001	0.896	0.002
IR ND (°)	69.5 ± 14.2	68.2 ± 14.5	−1.9	70.8 ± 13.9	69.5 ± 12.7	−1.8	0.001	0.059	1.000
ER D (°)	149.4 ± 12.3	153.5 ± 11.3	2.7	151.2 ± 13.3	156.7 ± 11.9	3.6	<0.001	0.003	0.032
ER ND (°)	135.7 ± 10.2	134.7 ± 10.3	−0.7	136.7 ± 10.3	137.4 ± 7.8	0.5	0.798	0.006	0.101
TROM D (°)	205.4 ± 19.4	206.3 ± 18.7	0.4	207.9 ± 20.3	208.7 ± 19.5	0.4	0.065	0.003	0.919
TROM ND (°)	205.2 ± 18.1	202.9 ± 18.4	−1.1	207.6 ± 18.0	206.9 ± 16.5	−0.3	0.045	0.001	0.112

IR = shoulder internal rotation; ER = shoulder external rotation; D = dominant side; ND = non-dominant side; ROM = range of motion; TROM = total range of motion.

**Table 4:** Data of female players showing shoulder strength and range of motion (ROM) measures at baseline and before or after the tennis training session.

	Before Tennis			After Tennis			*p*-Value		
Variables	Pre	Post	Δ%	Pre	Post	Δ%	Time Effects	Condition Effects	Interaction Effects
**Shoulder Strength**									
IR D Strength (N·m^−1^)	148.4 ± 29.1	138.3 ± 28.9	−6.8	150.4 ± 25.8	120.4 ± 21.2	−20	<0.001	0.009	<0.001
IR D Strength (N·m^−1^·kg^−1^)	2.7 ± 0.4	2.5 ± 0.4	−7.4	2.7 ± 0.4	2.2 ± 0.3	−18.5	<0.001	0.011	<0.001
ER D Strength (N·m^−1^)	94.8 ± 13.9	92.5 ± 10.8	−2.4	95.3 ± 12.0	90.4 ± 10.2	−5.1	0.07	0.496	0.051
ER D Strength (N·m^−1^·kg^−1^)	1.7 ± 0.3	1.7 ± 0.2	0	1.7 ± 0.2	1.6 ± 0.2	−5.9	0.07	0.443	0.038
IR ND Strength (N·m^−1^)	127.3 ± 29.9	122.6 ± 28.2	−3.7	128.0 ± 29.7	107.4 ± 39.1	−16.0	0.03	0.250	0.146
IR ND Strength (N·m^−1^·kg^−1^)	2.3 ± 0.4	2.2 ± 0.4	−4.4	2.3 ± 0.4	1.9 ± 0.7	−17.4	0.040	0.261	0.163
ER ND Strength (N·m^−1^)	94.6 ± 17.0	93.3 ± 18.0	−1.4	95.9 ± 16.8	95.6 ± 15.9	−0.3	0.381	0.180	0.146
ER ND Strength (N·m^−1^·kg^−1^)	1.7 ± 0.2	1.7 ± 0.2	0	1.7 ± 0.2	1.7 ± 0.2	0	0.352	0.137	0.117
**Shoulder ROM**									
IR D (°)	59.1 ± 10.2	57.9 ± 10.0	−2.0	60.4 ± 10.4	56.8 ± 10.1	−6.0	<0.001	0.865	<0.001
IR ND (°)	68.4 ± 13.6	67.4 ± 13	−1.5	69.5 ± 14.3	69.3 ± 13.4	−0.3	0.293	0.005	0.022
ER D (°)	152.5 ± 13.8	156.0 ± 13.0	2.3	153.3 ± 13.6	158.8 ± 12.5	3.6	<0.001	0.003	0.001
ER ND (°)	137.3 ± 13.9	135.6 ± 13.2	−1.2	138.9 ± 14.9	137.2 ± 13.9	−1.2	0.096	0.014	0.972
TROM D (°)	211.6 ± 18.9	213.9 ± 17.4	1.1	213.7 ± 17.8	215.6 ± 16.8	0.9	0.011	0.017	0.445
TROM ND (°)	205.7 ± 20.2	203.0 ± 18.5	−1.3	208.4 ± 22.2	206.5 ± 19.9	−0.9	0.051	0.004	0.416

IR = shoulder internal rotation; ER = shoulder external rotation; D = dominant side; ND = non-dominant side; ROM = range of motion; TROM = total range of motion.

## Data Availability

The data presented in this study are available upon request from the corresponding author. The data are not publicly available due to privacy restrictions from the tennis federation.
